# Companion animal adoption and relinquishment during the COVID-19 pandemic: Households with children at greatest risk of relinquishing a cat or dog

**DOI:** 10.1017/awf.2023.77

**Published:** 2023-08-18

**Authors:** Grace A Carroll, Alice Torjussen, Catherine Reeve

**Affiliations:** 1 Animal Behaviour Centre, School of Psychology, Queens University Belfast; 2 University of Sussex

**Keywords:** animal welfare, cat, children, dog, COVID-19, relinquishment

## Abstract

Understanding the factors associated with companion animal relinquishment is key in safeguarding animal welfare and human well-being. The aims of this study were to assess the effect of demographic variables on risk of relinquishment of cats and dogs during the COVID-19 pandemic, and to report characteristics of those that relinquished a cat or dog, and the experience of said relinquishment process. A series of surveys were administered to pet owners (n = 3,945) across several countries including the UK, USA, Canada, Italy, Spain and France. In total, n = 1,324 reported having acquired their cat or dog via online means. There was no association between online source (search engines, breeder websites, rescue websites, online ad sites and social media) and relinquishment status (NCR1 [Never Considered Relinquishment] compared to CR_R [Considering Relinquishment or already Relinquished]. More participants from the USA considered or already had relinquished their cat or dog compared to the UK and Italy. Of those that have already given up their pet, 76.2% agreed that it was an emotionally difficult decision, while 100% agreed that it was, logically, the correct decision. Demographic characteristics in those that reported considering relinquishment or that had already relinquished (CR_R; n = 146) were compared to a comparison group that had never considered relinquishing their pet (NCR2; n = 193). Being a male-gendered pet-owner and a younger pet age increased the risk of relinquishment. Cats and dogs from households with children were 4.6 times more likely to consider or have already relinquished a cat or dog compared to those from households without children. Further research is needed to explore risk of relinquishment of cats and dogs when children are present in the home.

## Introduction

In recent years, the nature of pet-ownership has changed in a number of ways. For example, COVID-19 led to an increased demand for cats and dogs (Morgan *et al.* 2020; Ho *et al.* 2021; Ng *et al.* 2021). During lockdown periods, pet owners spent more time with their companion animals than usual, increasingly relying on them to alleviate loneliness and provide a source of support during times of social isolation (Morgan *et al.* 2020; Jezierski *et al.* 2021). Changes to daily routines for companion animals and their owners were also seen during lockdown periods (Vincent *et al.* 2020; Christley et al. 2021; Holland *et al.* 2021). In addition, pet acquisition and relinquishment moved increasingly online (Hazel *et al.* 2018), with the COVID-19 pandemic being one influence on increasing online searches for pets (Siettou 2021). With online pet acquisition comes a number of problems including lack of regulation, trans-national crime and impulse buying (Maher & Wyatt 2019).

The proportion of cats and dogs being acquired online continues to rise. For example, Carroll *et al.* (2022) found that, of 3,945 current or previous dog and cat owners sampled in 2020, 39.0 and 26.6%, respectively, acquired their pet online. From data collected in early 2022, the 2022 PAWS report found that 63% of dogs and 43% of cats were acquired from an online source (People’s Dispensary for Sick Animals [PDSA] 2022). Most cats sourced online were found on rescue centre websites while most dogs were found on online advertising sites, such as Gumtree and Pets4Homes. Carroll *et al.* (2022) found a trend for greater likelihood of considering giving up, or giving a pet up, for those purchased online. However, risk of relinquishment according to online source requires further research. For example, cats and dogs can be sold in closed groups on social media, making them difficult to regulate and monitor. Furthermore, most online advertisement sites are unregulated, with poor uptake of voluntary standards where they do exist (EU Dog and Cat Alliance 2017). In contrast, cats and dogs advertised online by animal rescues and shelters will be subject to the same checks and requirements as those acquired off-line. For example, many animal rescues and charities offer pre- and post-adoption support (Hazel *et al.* 2018; Powell *et al.* 2021) such as pre-adoption talks (e.g. Dog’s Trust 2022) and behaviour and training services (e.g. Blue Cross 2023). Considering this, it is possible that risk of relinquishment will vary according to the online platform used to source companion animals.

The effect of the COVID-19 pandemic also varied across countries, with variation in mortality, restrictions, and level of adherence to public health measures (Karanikolo & McKee 2020; Margraf *et al.* 2021). Furthermore, differences in levels of and reasons for relinquishment can vary between communities within countries (Dolan *et al.* 2015). To date, much research on the effects of COVID-19 on pets and their owners has been conducted within a single country (Appiah *et al.* 2022). However, given the fluctuating effects of COVID-19, in addition to cultural variances, differences in relinquishment of cats and dogs between countries require further exploration. For the purposes of the current study, relinquishment will refer to all scenarios whereby individuals give up a cat or dog (for a discussion of this, see Carroll *et al.* 2022).

A lot is known about *why* people relinquish their pets, with a variety of factors influencing this decision from behavioural problems, pet owner expectations, housing-related issues, allergies, financial reasons and family composition and dynamics (Deleeuw 2010; Protopopova & Gunter, 2017; Applebaum *et al.* 2020; Jensen *et al.* 2020; Carroll *et al.* 2022). However, less is known about how relinquishers and those considering relinquishment differ from those that have never considered giving up their companion animal. A small number of previous studies have explored relinquisher characteristics compared to a comparison group. For example, New *et al.* (2000) interviewed cat and dog owners in 12 US shelters that wished to relinquish their pet. Demographic information included pet sex and age, acquisition source, and length of ownership. New *et al.* (2000) also distributed a mail survey to a comparison group of households that had a cat or dog. Several factors were more commonly seen in the relinquisher group, including the animal being unneutered, a short ownership period, and being a male pet owner. However, approximately half of the chosen households were selected for the mail survey because at least one pet had left the household in the last year. Therefore, the comparison group was not representative of the general pet owner population, most of which do not relinquish. Dolan *et al.* (2015) assessed demographic factors associated with dog relinquishment in a low-income community in the USA; those that attended a shelter to relinquish their pet were compared to a group of pet owners that were attending a low-cost spaying and neutering service. Factors including renting versus being a home-owner, being male, and stress within the home, were more commonly seen in the relinquisher group compared to the comparison group. More recently, Duarte Cardoso *et al.* (2022) compared 36 Portuguese relinquishers to 36 non-relinquishers of cats and dogs and found that those with children in the home and those living in apartments were at greater risk of relinquishment.

In addition, a lesser-studied topic is the experience of relinquishment from the owners’ perspective (Powell *et al.* 2022). Less recent studies suggest that the relinquishment process is difficult, with owners struggling with the decision to relinquish for a long time, viewing it as a last resort, and a very difficult decision to make (DiGiacomco *et al.* 1998, Shore 2003). However, there is little recent research on this topic.

This paper is one in a series of publications that form part of a larger project, ‘CAARP’ (Companion Animal Adoption and Relinquishment during the Pandemic), which seeks to understand adoption and relinquishment of cats and dogs across several countries from the perspective of pet owners, shelter staff, and from shelter records, employing a mixture of qualitative and quantitative approaches to data collection.

The aims of the current study were to:

- Assess the effect of online source and country of residence on risk of relinquishment of cats and dogs during the COVID-19 pandemic;
- Report characteristics of those that relinquished a cat or dog, and the experience of the relinquishment process; and
- Compare demographic characteristics of those that have given up, or have considered giving up their pets, to a comparison group of cat and dog owners.

## Materials and methods

**Figure 1.**
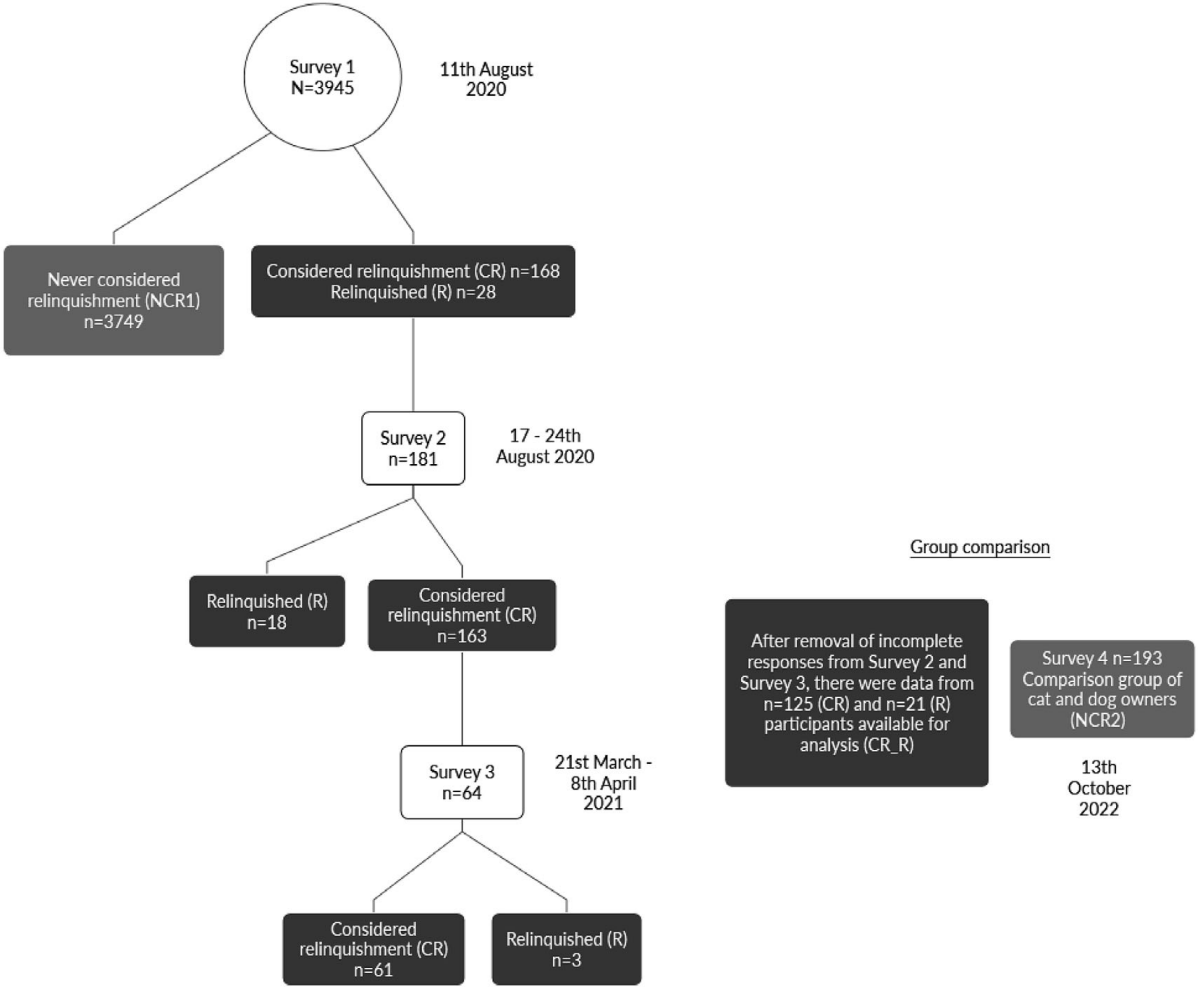
A visualisation of each stage of participant recruitment.

In a previous study, pet owners were surveyed in a cross-sectional study to assess the prevalence of self-reported relinquishment of cats and dogs during the COVID-19 pandemic (see Carroll *et al.* 2022). Briefly, 4,000 individuals were recruited (50% male sex) via Prolific Academic® and answered a range of demographic questions and questions on the cat or dog that they had most recently acquired. There were n = 3,945 usable responses. Those that reported having considered giving up a pet, and those that reported having already done so, were invited to complete a more detailed questionnaire (n = 181 responses) that collected demographic information (e.g. age, gender, employment status), living arrangements (e.g. number of children/adults in the home, home-ownership status), COVID-19 restrictions in place at the time of completion, the experience of ownership during the pandemic, and the experience of the relinquishment process (if applicable). Those that had considered relinquishment were surveyed again (survey 3) seven months later to assess relinquishment over time, with participants answering the same questions as they did for survey 2 (n = 64 responses). Finally, in order to allow for a comparison of demographic variables between Considered relinquishment (CR)/Relinquishers (R) and those that have never considered giving up a pet, survey 4 (a refined version of surveys 2 and 3) was distributed via Prolific Academic® to a comparison group of participants. For clarity, the recruitment process at each stage of data collection is visualised in Figure 1.

### Online source of acquisition

In survey 1, the source of the cat or dog was reported by participants (such as ‘shelter’, ‘breeder’ or ‘given as a gift’), and whether the animal was first sourced from an online advertisement. Participants that acquired their cat or dog via an online source were asked to provide the website used to source the animal. Carroll *et al.* (2022) assessed the source of acquisition (online: yes/no), finding a trend for greater likelihood of considering giving up or giving a pet up in those acquired from an online source. However, the effect of online source was not explored. In the current study, data collected on the online source of cats and dogs in survey 1 were classified into six categories based on free-text responses from participants: ‘search engine’, ‘breeder website’, ‘rescue website’, ‘online ad site’, ‘social media’, and ‘unknown’, where the written responses could not be deciphered, or the participant did not know, or could not remember, the online source.

### Country of residence

In survey 1, data were collected from a number of countries (see Carroll *et al.* 2022). However, between-country differences in relinquishment were not assessed. In the current study, countries with a participant sample of at least n = 100 participants were included in an analysis of relinquishment status by country. Five countries met this criterion: the UK, USA, Spain, Italy, and Canada.

### Characteristics of those that relinquished a cat or dog, and the experience of the relinquishment process

Participants that reported having considered relinquishment or having relinquished a pet in survey 1 (considered relinquishment: n = 163, relinquished: n = 28) were invited to complete a more detailed questionnaire, survey 2. Data collection for survey 2 was carried out between the 17th and 24th of August 2020. A total of 18 responses were received from those that had already relinquished. Seven months later, survey 3 was sent to those that have considered relinquishment to assess ownership status once more. Data collection for survey 3 were carried out between the 31st March and 8th April 2021. Three participants reporting having relinquished their cat or dog in the intervening months between survey 2 and survey 3. Therefore, information on the relinquishment process was available for a total of 21 participants.

### Comparison group analysis

Detailed demographic information was collected for those that considered relinquishment (CR) and have relinquished (R) in surveys 2 and 3. However, it was not possible to survey all 3,945 participants from survey 1 in detail, due to financial constraints. Therefore, a comparison group of dog and cat owners were surveyed via Prolific Academic®, using a refined version of survey 2 and 3. This allowed for an assessment of differences in demographic factors such as dwelling type, home ownership status and number of children in the home, between those who have never considered relinquishment (NCR2) and those that have considered it or have already given up their cat or dog (CR/R) (see Figure 1). A number of screening criteria were applied in recruiting the comparison group (NCR2) using the Prolific Academic® screening tools; a balance of male- and female-sexed participants were recruited. Prolific Academic® pre-screened respondents by using the question: ‘What sex were you assigned at birth, such as on an original birth certificate?’ Gender was then reported by participants within the survey, and it was self-reported gender, rather than biological sex, that was subsequently analysed. Participants were required to be resident in the UK, USA, Ireland, Spain, Italy, France, Canada or Australia to mirror the country of residence for the original sample of participants (Carroll *et al.* 2022). Western countries, where pet-keeping is well-established, were selected for this study. Participants were also required to be cat or dog owners, and those that had taken part in previous parts of the study were excluded from participation. Comparison group data were collected on the 13th October 2022.

### Statistical analysis

#### Online source of acquisition

Of the 3,945 participants that completed survey 1, n = 1,247 of Never Considered Relinquishment [NCR1] participants, n = 70 of Considered Relinquishment [CR] participants and n = 7 of Have Relinquished [R] participants acquired their cat or dog via an online source (for more details on the variables explored, see Carroll *et al.* 2022). Due to the small sample of relinquishers, relinquishment category was assessed as a binary variable (Never Considered Relinquishment [NCR1]/Considered or Have Relinquished [CR_R]) in a 2 × 6 Pearson’s Chi-squared test. A 2 × 6 Pearson’s Chi squared was also used to explore between-species (dog or cat) differences in the online source of acquisition. Descriptive statistics were used to explore the percentage of those that acquired their pet via online means for the following six source types: Adopted from a shelter/rehoming organisation; Purchased from a breeder; As a gift; Directly from someone that needed to find a new home for their cat or dog; Directly from someone that was seeking temporary care for their cat or dog; or The cat or dog was found as a stray. ‘Other’ sources were excluded from this analysis.

#### Country of residence

A 2 × 5 Pearson’s Chi-squared test was used to assess the relationship between relinquishment status (Never Considered Relinquishment [NCR1], Considered relinquishment or Have Relinquished [CR_R] and country of residence.

#### Characteristics of those that relinquished a cat or dog, and the experience of the relinquishment process

Descriptive statistics were used to explore relinquishers’ experience of pet ownership, relinquishment by country of residence, and to assess the process of giving up a pet.

#### Comparison group analysis

Descriptive statistics were used to analyse the demographic information. After carrying out exploratory analyses, a binary logistic regression was used to assess the Independent Variables: Home-ownership status (rent/own), Pet-owner gender (male/female), ‘Pet age’ (continuous) and ‘children in the home’ (yes/no), on the Dependant Variable = ‘Relinquishment status’ (yes/no). Variables were entered using backward selection. ‘Number of children’ was transformed into a binary variable due to the low number of participants reporting that they had three or more children in the home, creating a new variable ‘Children in the home’ (yes/no). ‘Dwelling type’ was initially included in the model. However, due to a suppressor effect occurring between ‘Dwelling type’, ‘Ownership status’ and the outcome variable, ‘Dwelling type’ was removed from the model (Guinn 2019).

SPSS version 29 was used for all analyses.

### Ethical considerations

This study was approved by Queen’s University Belfast Faculty Research Ethics Committee (EPS 20_111).

## Results

### Online source

#### Online acquisition for each type of source

The number of participants that reported originally sourcing their cat or dog online via the various acquisition sources can be seen in Table 1.

**Table 1.** The number of participants sourced their pet online for the various source types

	Online	
Source type*	Yes (%)	No (%)
Purchases from a breeder	52.5	47.5
Seeking temporary care	31.3	68.8
Needed new home	29.8	70.2
Adopted from shelter/rehoming organisation	28.6	71.4
Other	21.2	78.8
As a gift	10.1	89.9
Found as a stray	2.4	97.6

* Purchased from a breeder; Directly from someone that was seeking temporary care for their cat or dog; Directly from someone that needed to find a new home for their cat or dog; Adopted from a shelter/rehoming organisation; Other; As a gift; The cat or dog was found as a stray.

#### Online source by companion animal species

In total, 66% of pets acquired online were dogs and 34% cats. There was a significant association between the online source of acquisition and pet species (cat or dog), χ^2^ = 45.303 (5); *P* < 0.001. Cramer’s V = 0.41, indicating a large effect size. Bonferroni adjustment was used on all pair-wise comparisons (12 groups). An adjusted significance threshold of *P* = 0.0042 was set. More dogs were acquired via breeder websites (*P* = 0.0032) and online ad sites, compared to cats (*P* = 0.0037), while more cats were acquired from rescue websites than dogs (*P* < 0.0001). Species differences for acquisition from social media did not reach the adjusted threshold for significance (*P* = 0.0078). In addition, there was no difference by species for search engines and unknown online sources (*P* < 0.05). See Table 2 for the percentage of dogs and cats acquired from the various online sources.

**Table 2.** Online source according to pet species

	Species*	
Online source	Cat (%)	Dog (%)
Online ad site	40.6	49.0
Rescue website	23.1	12.5
Social media	17.7	12.4
Unknown	14.4	17.3
Search engine	2.4	2.7
Breeder website	1.8	6.2

* n = 1,324 reported having acquired their cat or dog via online means (n = 874 dogs, n = 450 cats)

### The effect of online source on relinquishment status

There was no association between online source and relinquishment status (NCR1 compared to CR_R), χ^2^ = 8.255 (5); *P* = 0.143. See Table 3 for the percentage of participants that reported acquiring their pets from the various online sources.

**Table 3.** Online sources of pet acquisition by relinquishment status

	Relinquishment status*		
Online source	NCR1 (%)	CR (%)	R (%)
Online ad site	46.0	51.4	28.6
Rescue website	16.3	12.9	14.3
Unknown	16.0	17.1	42.9
Social media	14.6	8.6	0.0
Search engine	2.4	5.7	14.3
Breeder website	4.7	4.3	0.0

* Of those that acquired their pet via an online source; Never considered relinquishment (NCR1) (n = 1,247), Considered relinquishment (CR) (n = 70), Have relinquished (R) (n = 7)

### Country of residence

In total, 9.9% in the USA, 6.4% in Canada, 5.8% in Spain, 4.4% in Italy and 3.0% of those residing in the UK, considered or already had relinquished their cat or dog (CR_R). There was a significant association between relinquishment status and country of residence, χ^2^ = 64.77 (4); *P* < 0.001, Cramer’s V = 0.26, indicating a large effect size. Bonferroni adjustment was used on all pair-wise comparisons (eight comparisons: UK and USA were compared to each other, Italy, Spain and Canada). An adjusted significance threshold of *P* < 0.006 was set. Significantly more participants from the USA (9.9%) considered or already had relinquished their cat or dog than in the UK (3.0%; *P* < 0.006) and Italy (4.4%; *P* = 0.003). No other country pair reached the adjusted threshold for significance (*P* < 0.006).

### The process of giving up a pet

Of those that had already given up their pet, 76.2% were dogs (n =16) and 23.8% were cats (n = 5). A total of 57.1% of relinquishers were male-gendered (n =12) and 42.9% were female-gendered (n = 9). In relation to country of residence, 61.9% of relinquishers were in the USA (n = 13), 23.8% in the UK (n = 5) and 14.4% (n = 3) were from other countries (Italy, France and Canada). In total, 90.5% (n = 19) were the primary carer of the relinquished animal. For 63.2% of participants, this was the first dog or cat they were responsible for. See Table 3 for a summary of the level of agreement with the following statements: ‘Giving up my pet was an emotionally difficult decision’ and ‘Giving up my pet was logically the correct decision.’

Six participants provided more detail on their reason for relinquishment. Two cited financial concerns (e.g. *“My partner was made redundant from her job and we could no longer afford to keep our dog and pay our bills”*) and another cited behavioural concerns *(“I could not handle its violent nature anymore, it was getting very aggressive so I had to do the needful to avoid putting myself and others in danger*”). Other participants’ reasons for relinquishment were varied, from pet age (*“The dog was getting quite old”*), transfer to another family member *(“My sister wanted to take care of him”*) and changes to family circumstances (*“We had another baby and it was too much for my wife”*).

### Comparison group analysis

#### Demographic characteristics

After removal of incomplete responses, there were n = 193 non-relinquishers (NCR2) and n = 146 considering or having already relinquished their cat or dog (CR_R [CR, n = 125, R = 21]). Overall, participants were from the UK (41.2%), USA (19.1%), Spain (11.0%), Italy (19.7%), Ireland (1.7%), France (1.4%), Canada (4.1%), Australia (1.2%), and 0.6% were from other countries. The mean age was 32.3 (± 9.9) years for CR_R participants, and 33.4 (± 11.3) for NCR2 participants. The mean pet age was 4.48 (± 3.5) years for R participants, and 6.07 (± 4.3) for NCR2 participants. See Table 4 for the percentage of CR_R and NCR2 participants within a set of demographic variables.

**Table 4.** The experience of relinquishment

	Statement	
Level of agreement	Giving up my pet was an emotionally difficult decision (%)	Giving up my pet was logically the correct decision (%)
Strongly agree	61.9	71.4
Agree	14.3	28.6
Neither agree nor disagree	9.5	0.0
Disagree	9.5	0.0
Strongly diagree	4.8	0.0

#### The effect of demographic characteristics on relinquishment status

For the binary logistic regression, the Dependent variable (CR_R or NCR2) and a set of Independent variables: ‘Dwelling type’ (four categories), Ownership status (rent/own), Pet-owner gender (male/female), ‘Pet age’ (continuous) and ‘Children in the home’ (yes/no), were entered in the model. The logistic regression model was statistically significant, χ^2^ (4) = 65.363; *P* < 0.001. The Hosmer and Lemeshow *P*-value indicated that the model was a good fit (*P* = 0.394). The Nagelkerke R square value indicated that 23.5% of the variation in the Dependent Variable was accounted for by the model. The predictive capacity of the model was 68.4%, reflecting an increase of 11.5% compared to the null model (56.9%). Male-gendered participants had a 49.6% increase in probability of relinquishment compared to females (*P* = 0.005); for every one-year increase in pet age, there was a 91.5% decreased probability of relinquishment (*P* = 0.006); and participants from households with children were 4.6 times more likely to consider or have already relinquished a cat or dog compared to those from households without children (*P* < 0.001). In addition, there was a trend towards renters having an increased probability of considering or having already relinquished a pet (*P* = 0.059).

## Discussion

In this study, we explored the effect of online source and country of residence on relinquishment of companion animals during the COVID-19 pandemic. In order to assess demographic variables associated with relinquishment, data from a comparisons group of dog and cat owners were compared to those of individuals that have considered, or already relinquished, a cat or dog.

### Online source

Overall, there was a difference in online source according to pet species. While more dogs were acquired via advertisement sites, this was the most common online acquisition source for both dogs and cats. More dogs were acquired via breeder websites compared to cats, while more cats were acquired from rescue websites than dogs. This finding is similar to those within the 2021 PAWS report (PDSA 2021) on UK pets, which found that dogs were most often acquired from breeders while cats were most often acquired from a rescue centre or rehoming centre.

The online source of pet acquisition did not vary between those that had never considered relinquishing a cat or dog, and those that had considered it or had already given up their pet. This suggests that no one online source is associated with increased likelihood of relinquishment. However, it is important to note that only seven participants that had relinquished a pet acquired their animal via online means, with 70 participants considering relinquishment having acquired their pet online. Nonetheless, this finding is somewhat surprising given the problems associated with regulation of online sales (Maher & Wyatt 2019). Some advertisement sites including Pets4Homes, Gumtree and Preloved have ascribed to the Pet Advertising Advisory Group (PAAG) minimum standards for online advertisements (Cat’s Protection 2021). Future research could assess risk of relinquishment between sites that do, and do not, follow these guidelines. This was not possible in the current study due to the relatively small sample of participants that had acquired their pet online and subsequently expressed the desire to give up their companion animal. Furthermore, the PAAG guidelines exist only in three of the countries assessed in the current study; the UK, Ireland, and more recently, Italy (EU Dog & Cat Alliance 2020). While most countries lack equivalent voluntary standards (Goncalves Costa *et al.* 2020), the number of countries ascribing to PAAG guidelines in the EU is, fortunately, on the increase (EU Dog & Cat Alliance 2020).

### Country of residence

Despite the number of UK residents in the original sample (survey 1: UK, n = 2,305, USA, n = 828), there were a greater number of participants from the USA that had considered giving up or had already given up their pet compared to the UK. In those that had already relinquished a pet, almost two-thirds were resident in the USA. Previous US-based studies have found a similar figure to that identified in the current study, with a 9.2% return rate to one USA shelter in a five-year retrospective study (Powell *et al.* 2022). Similarly, Hoffman *et al.* (2021) sampled over 10,000 participants from across the USA and found that 9% of cat and/or dog owners were considering relinquishment in next three months, while 12% of pet owners reported having rehomed a dog and/or cat between March 2020 and June 2021. While the impact of COVID-19 changed rapidly from country-to-country, the USA, UK and Italy were some of the worst affected countries in terms of human mortality (Matta *et al.* 2020). Despite this, relinquishment and consideration of relinquishment of companion animals were significantly lower in the UK and Italy relative to the US. Therefore, it is likely that factors other than COVID-19 play a role in between-country differences in dog and cat relinquishment. More research is needed to compare relinquishment figures between countries and to uncover potential reason for these differences.

### Species

Carroll *et al.* (2022) found that there were no species differences in relinquishment when those *considering* relinquishment were included in the analysis. When we look only at those that gave up their pet, the majority of these participants relinquished a dog (16/21). While the sample of relinquishers was small, this could suggest that the decision to relinquish is made more often for dogs than cats. Duarte Cardoso *et al.* (2022) assessed factors influencing relinquishment by comparing 36 relinquishers and 36 non-relinquishers from a sample of Portuguese cat and dog owners and, similar to the current study, found that 70% of relinquished pets were dogs (25/36). A recent report on UK pets (UK Pet Food, as cited in Palacios Rubio 2023) surveyed approximately 9,000 pet owners and found that in 2022, 47% of relinquished pets were dogs while 36% were cats. However, this difference could be due to the greater number of dogs kept as pets compared to cats. For example, UK- and USA-based surveys have consistently found that more households contain dogs than cats (Hawes *et al.* 2022; PFMA Pet Population 2022; Anderson *et al.* 2023). More research is needed to compare relinquishment rates between these species to identify reasons for variation in the number of animals being relinquished, while taking account of the popularity of each species as a companion animal.

### The relinquishment process

In the current study, 63.2% of relinquishers were first-time pet owners. Packer *et al.* (2021) found that 40.3% owners that acquired a puppy during the COVID-19 period had no prior experience of dog ownership, an increase from 33.3% of dog owners that acquired a puppy in 2019. Similarly, in their national survey of dog ownership in the UK, Anderson *et al.* (2023) found an increase in younger individuals acquiring a puppy and highlighted the need to assess the effect of age and ownership experience on risk of relinquishment. The high prevalence of first-time pet owners in the current study suggests that support is particularly important for new owners in acquiring a suitable pet, retaining that pet, or relinquishing the animal safely when required.

Most relinquishers agreed (‘agree’ or ‘strongly agree’) that giving up their pet was an emotionally difficult decision. However, 14.3% disagreed with this statement, demonstrating that that the relinquishment experience varies from person-to-person. Kwan and Bain (2013) compared attachment levels in dog owners relinquishing to a shelter with those visiting a vaccine clinic and found that relinquishers were less attached to their dogs than the comparison group of pet owners. In contrast, Dolan *et al.* (2015) found that all dog owners were strongly attached to their pet, regardless of relinquishment status and Shore (2005) reported that 56.6% of relinquishers rated the process on a ten-point scale as a ten (‘very difficult’). In the current study, all participants believed that giving up their pet was logically the correct decision. This could indicate that the decision to relinquish was well-thought-out. Indeed, the decision to relinquish can take weeks and months of consideration, with owners trying to avoid relinquishment or find an alternative home themselves prior to approaching an animal shelter (DiGiacomo *et al.* 1998; Sharkin & Ruff 2011; Weiss *et al.* 2014). The current findings support previous suggestions that giving up a pet is indeed difficult for the pet owner. In addition to providing support to *avoid* relinquishment, support should also be available for those that go ahead with this decision.

### Group comparison

#### Household composition

Several differences were found between relinquishers (actual or considered) and the comparison sample of pet-owners. As identified previously, male-gendered participants were at increased risk of relinquishment compared to females. Previous research has also shown a link between pet-owner gender and risk of relinquishment (New *et al.* 2000; Dolan *et al.* 2015, see Carroll *et al.* 2022 for discussion). The variable with the largest influence on risk of relinquishment was having children in the home, with households containing children being almost five times more likely to consider relinquishment or actually relinquish a pet than households without children. Spending time with pets during lockdown periods often acted as a coping strategy or stress buffer for children and young people (Applebaum *et al.* 2020; Charmaraman *et al.* 2022). Despite this, several studies suggest that having children in the home increases the risk of relinquishment and other problems. For example, Hoffman *et al.* (2021) found that, in a US sample, households with children were almost twice as likely to consider giving up their pet. Similarly, in a Portuguese study, Duarte Cardoso *et al.* (2022) found that 36% of relinquisher households had children under 12 years of age and 53% had children under 18 years of age, compared to 14 and 19% of non-relinquishing households, respectively. In terms of potential reason for this, Powdrill-Wells et al. (2021) found aggression around children to be a common reason for dog relinquishment requests at one UK rehoming organisation. Furthermore, attendance at paediatric emergency department for dog bites increased three-fold during the COVID-19 pandemic at times where children were required to stay at home (Tulloch *et al.* 2021). Therefore, aggression towards children may be an important contributor to this. Pre-COVID, Kwan and Bain (2013) found that relinquishing owners had more children in the home than a comparison group of pet-owners. Thus, the reasons for relinquishment of pets when children are present may be multifaceted and not solely related to COVID-19 restrictions. Qualitative research may be particularly useful in assessing why having children in the home poses a problem in terms of retention of cats and dogs. Animal rescues and shelters should ensure that families with children are aware of any potential risks or difficulties that may be encountered when adopting a new pet, with post-adoption support being one avenue for intervention.

### Animal age

In the current study, younger animals were at increased risk of relinquishment. This is in line with previous relinquishment research in the USA, UK and Australia (New *et al.* 2000; Casey *et al.* 2009; Arbe Montoya *et al.* 2017). However, others have found older animals to be at increased risk (Casey *et al.* 2009; Kwan & Bain 2013). Indeed, in the current study, one participant mentioned *“The dog was getting quite old”* as a reason for relinquishment. On a positive note, younger animals are often adopted more quickly (Brown & Morgan 2015; Fatjo *et al.* 2015). Therefore, younger animals that are relinquished have a good chance of being successfully rehomed.

### Home-ownership status

There was a trend towards increased risk of relinquishment in individuals that rent their homes compared to home-owners. As can be seen in Table 5, numerically, there is little difference in dwelling type between relinquishers and the comparison group. Indeed, more relinquishers (actual or considered) lived in houses with gardens (5.3%) compared to non-relinquishers (3.6%). It therefore appears that home ownership status matters more than space availability. However, Weiss *et al.* (2015) found that the most common housing-related reasons for relinquishment in US households were landlords not allowing pets, followed by inadequate space. Renters face a number of obstacles to pet-ownership including pet rent and other fees, limits on the number of pets, species, size and breed restrictions, and outright bans on pet-keeping (Shore *et al.* 2003; Graham & Rock 2019; Rose *et al.* 2023). These factors may mean that renters are at greater risk of relinquishing a cat or dog. However, in the current study, this variable did not reach the significance threshold. The risk of relinquishment of pets in those in rented housing needs to be explored in greater detail, potentially using qualitative methods to dig deeper into the challenges faced by renters and those living in different types of accommodation.

**Table 5.** The percentage of CR_R and NCR2 participants for each demographic variable

		Relinquishment status	
Demographic variable		CR_R (%)	NCR2 (%)
Species			
	Cat	41.7	47.9
	Dog	58.3	52.1
Gender			
	Male	66.7	49.7
	Female	33.3	50.3
Dwelling type			
	House with garden	65.6	56.3
	House with no garden	5.3	3.6
	Apartment with garden	17.2	15.1
	Apartment with no garden	11.9	25.0
Home ownership			
	Own	58.3	69.6
	Rent	41.7	30.4
Children in the home			
	0 children	35.1	70.1
	1 child	27.8	13.9
	2 children	32.5	11.9
	3 or 4 children	4.6	4.1
Adults in the home			
	1 adult	7.9	12.9
	2 adults	49.0	47.4
	3 adults	21.9	17.0
	4 adults	10.6	18.6
	5 or more adults	10.6	4.1
Other pets in the home			
	Yes	43.7	40.2
	No	56.3	59.8
Martial status			
	Married/partner	65.6	54.1
	Single	31.1	41.8
	Seperated	0.7	2.1
	Divorced	2.6	2.1
Education			
	Secondary school	19.9	32.5
	College/University degree	51.0	43.8
	Master’s degree	23.8	20.6
	Doctoral degree	2.0	1.0
	Other professional graduate/PG degree	3.3	2.1
Employment			
	Employed	69.5	63.9
	Not working	14.6	14.9
	Student	2.0	2.1
	Retired	13.9	19.1
Illness			
	Mental	5.3	7.2
	Physical	5.3	7.2
Reason for acquisition*			
	Companionship	94.0	96.9
	Use as a service animal	5.3	0.0
	Training/Competitions	6.7	0.5
	Working animal	2.0	0
	Other^#^	3.9	5.1

* could select as many as apply, #other = gift, passed down, stray, pest deterrent. CR_R = Considered relinquishment (CR) or Have relinquished (R).

### Limitations

A limitation of this study was the relatively small number of participants that reported having given up a dog or cat. This may be due to low levels of relinquishment, but may also be a result of social desirability effects, whereby individuals are unwilling to admit to giving up their pet. Further research is needed to compare actual relinquishment and abandonment rates of cats and dogs throughout the COVID-19 pandemic from shelter and rescue data. This will give a clearer picture of the extent of the issue. The current study was also restricted to a small number of Western countries, with most participants coming from the UK and the USA. It would be of benefit to expand research to include a greater number of countries in the future. A final limitation to consider is the time gap between data collection on those that have considered relinquishment, or have already relinquished, and the comparison group of dog and cat owners. This may have impacted the results as the effects of COVID-19 changed across time.

### Animal welfare implications

The current study identified several demographic factors associated with risk of relinquishment of cats and dogs across several first-world countries. Male pet-owners, households with children, and younger pets were found to be at greater risk of relinquishment. This information can be used to improve our understanding of influences on the decisions to relinquish a pet and can inform interventions to avoid such a situation. Most notably, families with children in the home may benefit from additional support in the pre- and post-adoption periods.

## Conclusion

Despite concerns surrounding the increase in online acquisition of pets, no one online source was associated with increased risk of relinquishment of cats and dogs. Pet-owners in the USA relinquished animals at the highest rate of any of the surveyed countries. For those that have relinquished a cat or dog, the decision to relinquish was difficult for most pet-owners but logically the correct decision for all. Having children in the home posed the biggest risk in terms of the desire to relinquish a cat or dog, while being a male owner, and having a younger pet also increased the risk of relinquishment. Further research is needed to assess differences in relinquishment risk between countries, and risk of relinquishment when children are present in the home.
